# Roles of extended human papillomavirus genotyping and multiple infections in early detection of cervical precancer and cancer and HPV vaccination

**DOI:** 10.1186/s12885-021-09126-3

**Published:** 2022-01-06

**Authors:** Fangbin Song, Peisha Yan, Xia Huang, Chun Wang, Hui Du, Xinfeng Qu, Ruifang Wu

**Affiliations:** 1 Department of Obstetrics and Gynecology, Peking University Shenzhen Hospital, Shenzhen, 518036 Guangdong P. R. China; 2 Institute of Obstetrics and Gynecology, Shenzhen PKU-HKUST Medical Center, Shenzhen, 518036 Guangdong P. R. China; 3 Shenzhen Key Laboratory on Technology for Early Diagnosis of Major Gynecological Diseases, Shenzhen, 518036 Guangdong P. R. China; 4 Sanming Project of Medicine in Shenzhen, Peking University Shenzhen Hospital, Shenzhen, 518036 Guangdong China

**Keywords:** Human papillomavirus, Genotype, Cervical intraepithelial neoplasia, Cervical cancer, Screening, Vaccination

## Abstract

**Background:**

The aim of the study was to investigate the risk of human papillomavirus (HPV) genotyping particularly vaccine genotypes and multiple infections for cervical precancer and cancer, which might contribute to developing genotype-specific screening strategy and assessing potential effects of HPV vaccine.

**Methods:**

The HPV genotypes were identified using the Seq HPV assay on self-collected samples. Hierarchical ranking of each genotype was performed according to positive predictive value (PPV) for cervical intraepithelial neoplasia 2/3 or worse (CIN2+/CIN3+). Multivariate logistic regression model was used to estimate the odds ratios (ORs) with 95% confidence interval (CI) of CIN2+ according to multiplicity of types and vaccine types.

**Results:**

A total of 2811 HPV-positive women were analyzed. The five dominant HPV genotypes in high-grade lesions were 16/58/52/33/18. The overall ranking orders were HPV16/33/35/58/31/68/18/ 56/52/66/51/59/45/39 for CIN2+ and HPV16/33/31/58/45/66/52/18/35/56/51/68/59/39 for CIN3+. The risks of single infection versus co-infections with other types lower in the hierarchy having CIN2+ were not statistically significant for HPV16 (multiple infection vs. single infection: OR = 0.8, 95%CI = 0.6-1.1, *P* = 0.144) or other genotypes (*P* > 0.0036) after conservative Bonferroni correction. Whether HPV16 was present or not, the risks of single infection versus multiple infection with any number (2, ≥2, or ≥ 3) of types for CIN2+ were not significantly different. In addition, HPV31/33/45/52/58 covered by nonavalent vaccine added 27.5% of CIN2, 23.0% of CIN3, and 12.5% of cancer to the HPV16/18 genotyping. These genotype-groups were at significantly higher risks than genotypes not covered by nonavalent vaccine. Moreover, genotypes covered by nonavalent vaccine contributed to 85.2% of CIN2 lesions, 97.9% of CIN3 and 93.8% of cancers.

**Conclusions:**

Partial extended genotyping such as HPV33/31/58 but not multiplicity of HPV infections could serve as a promising triage for HPV-positive self-samples. Moreover, incidence rates of cervical cancer and precancer were substantial attributable to HPV genotypes covered by current nonavalent vaccination.

**Supplementary Information:**

The online version contains supplementary material available at 10.1186/s12885-021-09126-3.

## Background

Cervical cancer is a common malignant disease that threatens the health of women, caused 311,365 deaths worldwide in 2018 [1]. Efforts should be attached to further reduce the burden of cervical disease and eventually achieve the goal of eliminating cervical cancer [2]. High-risk human papillomavirus (hrHPV) was found to be a necessary cause of cervical cancer [3], leading to the development of HPV-based screening and vaccine for cervical cancer prevention and control. Fortunately, many of HPV infections cause minor cytology abnormalities progress to cervical precancers, and only a subset of precancers become invasive cancers [4, 5]. Information on type-specific risks for cervical diseases may help monitor effectiveness of HPV vaccine, and may aid in the individualized triage plans, particularly for HPV-based screening on self-samples [6].

Although current US guidelines recommend HPV16/18 genotyping as a triage option in HPV-positive women [7], HPV16/18 genotyping fails to detect cervical lesions associated with other genotypes. Whether extended genotypes (hrHPV genotypes except for HPV16/18) should be considered for triage or not is still well worth investigating. Recently, we showed in a large study that 75.8% of abnormal cytology and 50.9% HSIL cytology were attributed to other hrHPV infection among HPV-positive women, and 62.7%/43.9% of CIN2/CIN3+ were caused by other hrHPV infection over 3-year follow-up [8]. Moreover, the introduction of vaccines could lead to the eradication of HPV16/18 [9–11], better understanding of extended genotyping provides information for establishing favorable screening policies following the introduction of vaccines [12].

Genotype-specific reports often include information about multiple infections (more than one types of HPV infection) with the application of full genotyping assays. To our knowledge, the role and mechanisms of HPV coinfection in cervical carcinogenesis are still not fully understood [13]. Coinfections were reported more likely to have cytologic abnormality than those with single infections [14]. However, the histologic correlation and clinical significance of multiple infections remain debatable [15, 16]. Additionally, estimating the impact of a vaccine is difficult due to the presence of multiple infections.

HPV vaccine is a powerful tool in cervical cancer prevention [17]. Although three HPV vaccines are available in mainland China, none of them has been incorporated into the National Immunization Program yet. In addition, the current vaccines do not protect against all hrHPV types. With the approval and development of HPV vaccine in China, there is an urgent need for extensive studies to clarify cervical carcinogenesis of full genotypes, and to predict the potential efficacy of available vaccines on the reduce of cervical lesions since the ultimate goal of the vaccine is not to prevent HPV infection, but to prevent the occurrence of cervical cancers and precancers.

HPV testing done with a clinically validated PCR-based assay had similar accuracy on self-samples and clinician-samples in our and other large clinical trials [18–21]. Thus, HPV self-sampling could be used as a primary screening approach in routine screening to increase screening coverage. Based on a large cervical screening program using SeqHPV assay on self-samples, this study was aimed to (a) assess distribution of HPV genotypes in different histologic grades, and determine risks of individual genotypes for detection of cervical diseases, (b) to investigate the role of multiple HPV infections, (c) to evaluate potential impacts of available vaccines by risk determination of HPV genotypes covered by current vaccines, thus providing a basis for HPV vaccine implementation and cervical screening strategy.

## Methods

### Study population and design

Between Nov 2018 and Dec 2019, we conducted a population-based cervical screening project using HPV testing on self-collected samples as the primary screening, which was well-organized at 12 counties in Henan Province, Central China, with 187,000 non-pregnant women aged 30-64 years being screened. Large-scale cervical screening program was not performed in the past 3 years in these counties. This cervical screening program spans 3 years. Of the total 187,000 from 12 counties in the large cervical cancer screening program, we selected 3 counties including 73,699 women to carry out this prospective observational study. The three counties are connected geographically, and the secondary screening strategy of these three adjacent counties was different from that of other counties. Thus, the current study was nested into this large cervical screening program, a total of 73,699 women who consented for participation via signature on registration website from three adjacent counties were enrolled into this study (Enrolled women). More details were reported in our recent published articles [22, 23].

The study protocol was conformed to the Declaration of Helsinki, and the digital informed consent was approved by the Institutional Ethics Committee of Peking University Shenzhen Hospital (PUSH, No. 2018035) and local institutions based on the prior approval from Institutional Ethics Committee of BGI-Shenzhen for the digital informed consent form and its signature manner. Information that could identify individual participant was fully anonymized during or after data collection. The current analysis focuses on women with complete data on HPV genotyping and biopsy-based histologic results from this large population.

### Screening procedures

**Fig. 1:**
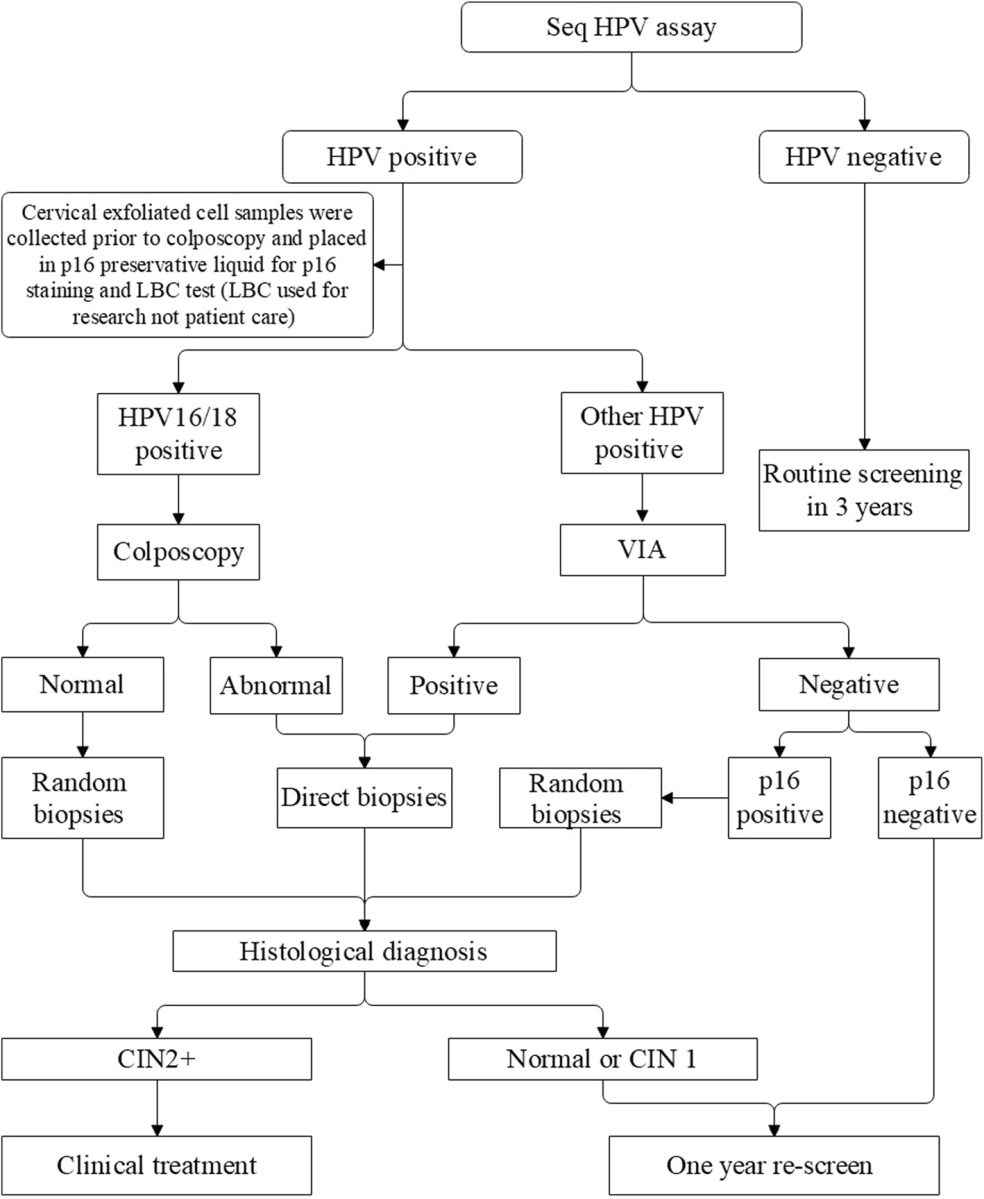
Flowchart of the screening protocol. VIA, visual inspection under acetic acid; LBC, liquid-based cytology. Genotyping for HPV16/18, VIA, and p16 immunostaining were used for triage sequentially

After successful registration for participation and information registration via the mobile device, eligible woman was asked to collect a cervicovaginal specimen with a cyto-brush by herself, following the instruction on a graph-text guide. Special instruction would be offered by the on-site provider if any woman had difficulty in understanding the sampling guide. The samples were rubbed on the solid media transport card (FTA card) by placing it in the middle of the application area and rolling it one full rotation, the self-collected samples were sent to the Center of BGI Health Clinical Laboratory, Wuhan, China for SeqHPV assay. Women with negative HPV result were advised to regular screening after 3 years, while those with positive HPV results were called back for triage and collected cervical samples with a cyto-brush before colposcopy or visual inspection under acetic acid (VIA) for p16^INK4a^ immuno-cytology and liquid based cytology (LBC) test, LBC was used for research purpose but not patient care. Before referral of subjects with HPV-positive results for colposcopy, the study group from PUSH provided training of all the management protocol procedures for local gynecologists and pathologists. Women were referred for colposcopy/biopsy if they were: (a) positive of HPV16 and/or 18; (b) positive for both other types and VIA; or (c) other HPV-positive, VIA negative but abnormal of p16 staining (Fig. 1). Patients with pathological diagnosis of cervical intraepithelial neoplasia 2 or worse (CIN2+) were recommended to be treated according to the clinical diagnosis and treatment procedures of PUSH (Fig. 1).

### HPV genotyping

All self-collected samples were prepared for SeqHPV assay (HPV genotyping based on sequencing, BGI Shenzhen, Shenzhen, China), an HPV genotyping assay using multiplex PCR and next generation sequencing [24]. The accuracy and reproducibility of Seq HPV assay for primary cervical cancer screening have been validated in SHENCCAST II [24] and CHIMUST [20] in comparison with the FDA-approved tests such as HC-2 and Cobas 4800 HPV assay. Moreover, SeqHPV assay has been approved by China Food and Drug Administration (CFDA). By designing a series of unique primers, the multiple index PCR system amplifies the approximately 150 bp of the HPV L1 gene with high-throughput capacities and type-specific output, and capable of processing greater than 4500 samples in 24 h [20, 24]. Seq HPV assay individually identifies 14 types of hrHPV (16, 18, 31, 33, 35, 39, 45, 51, 52, 56, 58, 59, 66, and 68) and two types of low-risk HPV (lrHPV, HPV6 and 11).

### Colposcopy-directed biopsy and histologic diagnoses

Colposcopy-directed biopsy was completed within 6 months after primary HPV screening according to a protocol modified from the quadrant-based Preventive Oncology International (POI) protocol [25]. According to the protocol, random biopsies would be taken at 2, 5, 8, and 11 o’clock for patients without visible lesion, while multiple biopsies would be taken at the VIA-indicated lesion site(s) plus the opposite quadrant of the transformation-zone for women with visible lesion(s). Histologic diagnoses were obtained according to the colposcopy-directed biopsy, and the highest diagnosis was recorded in women who had more than one tissue specimens (colposcopy orientation, random, or endocervical curettage). When it’s difficult to identify CIN2 and CIN3, p16 immunostaining was conducted. Histologic results were divided into normal (including cervicitis, and HPV infection without sign of CIN), CIN1, CIN2, CIN3 (including adenocarcinoma in situ, AIS), and cervical cancers (including squamous cell carcinoma, and adenocarcinoma). The slides were reviewed by pathologists of local hospital primarily and further confirmed by the senior gynecological pathologists from PUSH. Any discordant result between study pathologists and local pathologists was finalized by consensus review. Pathologists were blinded to LBC, p16, and HPV genotyping, but not to HPV positive outcome.

### Statistical analysis

Biopsy-confirmed CIN2+ (including CIN2/3, AIS and cervical cancers) and CIN3+ (including CIN3, AIS and cervical cancers) were used as the study endpoints. CIN2+ is compared to normal and CIN1; CIN3+ is compared to normal + CIN1 + CIN2. The Mantel-Haenszel Chi-square test was carried out to investigate any linear trend in proportions. HPV6 and HPV11 are considered low vs. hrHPV. Positive predictive values (PPVs) for CIN2+/CIN3+ were calculated to estimate the risk of disease for each hrHPV genotype, and hierarchical rankings of hrHPV genotypes for CIN2+/CIN3+ were formed based on sequentially maximizing the PPV for the new genotype, each preceding genotype was excluded when calculating the risk of the subsequent genotype [11]. The model assumes that the risk of disease in subjects co-infected multiple genotypes is determined by the highest-risk genotype. Cumulative sensitivity and specificity for increasing numbers of genotypes ordered by the hierarchy were calculated.

Since CIN2+ (including CIN2/3 and cancers) is the threshold of clinical treatment and has a greater number than CIN3+, vaccine type groups were calculated according to genotypes ordered by the hierarchy for CIN2+. The risks of CIN2+ in relation to type groups covered by vaccines, and the risks of multiple infection versus single infection by individual type were assessed by using multiple logistic regression model. Odds ratios (ORs) with 95% confidence interval (CI) were adjusted for potential confounders such as age, screening sites, and HPV infection pattern (Indicating three infection categories, including lrHPV, hrHPV, and hrHPV+lrHPV). A prior study indicates that co-infection with lrHPV interferes with the rate of progression to cervical cancer [26], thus lrHPV was included into the analysis of ORs. *P*-values from multiple comparisons were adjusted by conservative Bonferroni correction. Both hierarchical and proportional attribution models were used for the estimation of vaccine coverage for histologic diagnosis [27, 28]. Analyses were conducted using SPSS software (IBM Corporation, version 24.0) and Stata/SE 15.1 software. All analyses were two-sided, *P*-value < 0.05 was considered statistically significant.

## Results

### Characteristics of study population and patients including

**Fig. 2:**
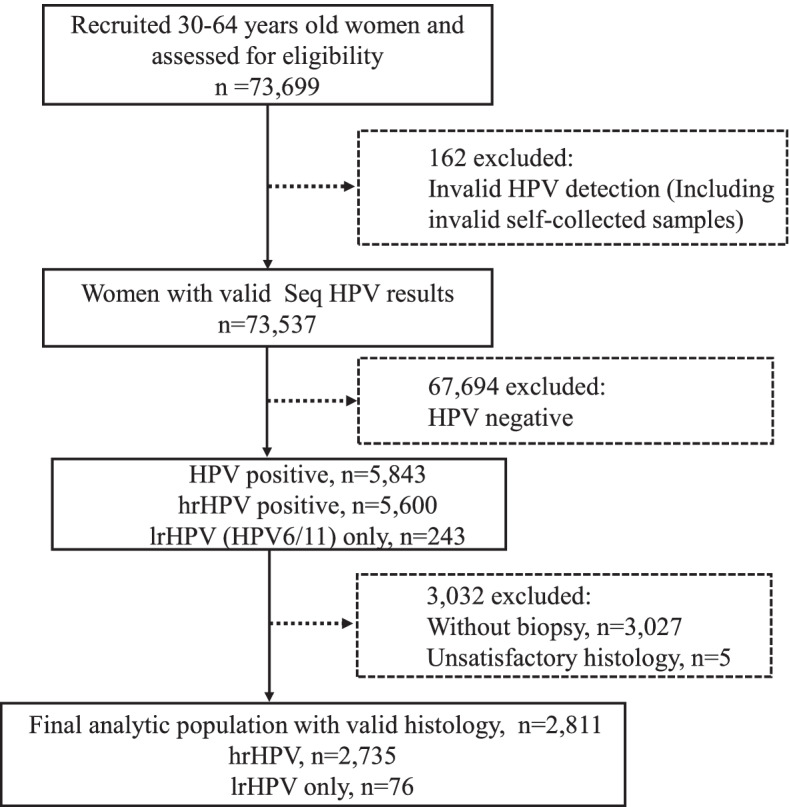
Flow diagram of study population selection. HPV, human papillomavirus; lrHPV, low-risk HPV; hrHPV, high-risk HPV

Overall, 7.95% (5843/73,537) and 7.62% (5600/73,537) women were detected to be HPV-positive and hrHPV-positive respectively, and 243 (0.33%) cases had “only lrHPV” infection (Fig. 2). A total of 3027 HPV positive women did not undergo colposcopy-directed biopsy, and 5 cases had an unsatisfactory histology were excluded. After excluding incomplete data, a total of 2811 women were eventually included in the analysis (Fig. 2). The median age of the study population was 48 years (range: 30 to 64 years). Among them, 2735 cases were hrHPV positive, and seventy-six cases had only lrHPV infection, and 2371 were diagnosed of ≤CIN1, 189 of CIN2, 235 of CIN3 (4 of AIS), and 16 of cancers (Table 1). The number of HPV types and that of hrHPV types were not associated with the severity of cervical lesions (HPV types, P_trend_ = 0.625; hrHPV types, P_trend_ = 0.803, Table 1).

**Table 1:** Baseline characteristics

Characteristics	Total	Histology, n (%)		
		≤CIN1	CIN2	CIN3+
**Total**	2811	2371	189	251
**Age (Y)**	47.9 ± 8.2	48.1 ± 8.2	46.8 ± 8.1	47.7 ± 8.9
30-45	1008	826 (81.9)	85 (8.4)	97 (9.6)
> 45	1803	1545 (85.7)	104 (5.8)	154 (8.5)
**Screen settings**				
County A	1212	1077 (88.9)	65 (5.4)	70 (5.8)
County B	832	679 (81.6)	64 (7.7)	89 (10.7)
County C	767	615 (80.2)	60 (7.8)	92 (12.0)
**HPV infection pattern**				
hrHPV	2735	2298 (84.0)	186 (6.8)	251 (9.2)
hrHPV+lrHPV	86	73 (84.9)	8 (9.3)	5 (5.8)
Single lrHPV	76	73 (96.1)	3 (3.9)	0 (0.0)
**No. of HPV types**				
1	2054	1743 (84.9)	122 (5.9)	189 (9.2)
2	569	470 (82.6)	52 (9.1)	47 (8.3)
≥ 3	188	158 (84.0)	15 (8.0)	15 (8.0)
**No. of hrHPV types**				
1	2031	1715 (84.4)	124 (6.1)	192 (9.5)
2	536	441 (82.3)	49 (9.1)	46 (8.6)
≥ 3	168	142 (84.5)	13 (7.7)	13 (7.7)

*Abbreviations*: *HPV* human papillomavirus, *lrHPV* low-risk HPV, *hrHPV* high-risk HPV, *CIN* cervical intraepithelial neoplasia, ≤CIN1 normal or CIN1, *CIN3+* cervical intraepithelial neoplasia 3 or worse

### Distribution of HPV genotypes and multiple HPV infection among different histologic grades

**Fig. 3:**
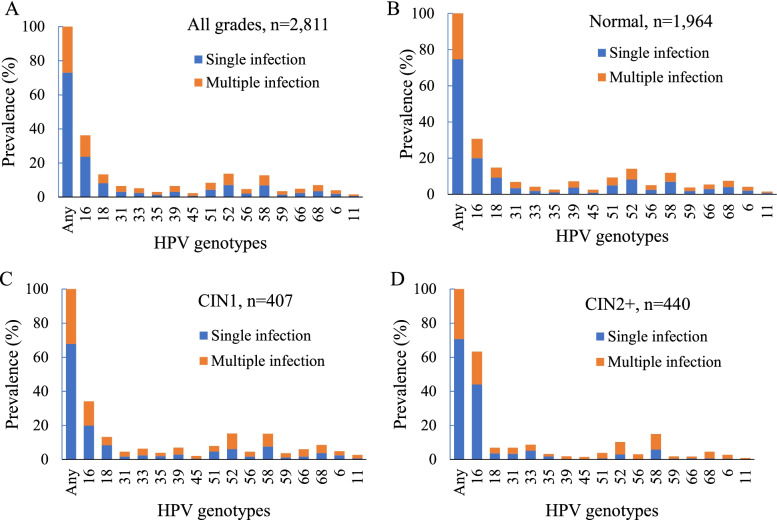
Prevalence of single and multiple infection among (**A**) all pathologic grades; (**B**) normal pathology; (**C**) CIN1; (**D**) CIN2+

In 2811 HPV positive women, HPV16 (36.3%), HPV52 (13.8%), HPV18 (13.4%), HPV58 (12.9%), and HPV51 (8.4%) were the five most common genotypes. In women within CIN1, the 5 most prevalent hrHPV types were HPV16 (34.2%), HPV52 (15.4%), HPV58 (15.2%), HPV18 (13.3%), and HPV68 (8.6%); while HPV16 (63.4%), HPV58 (15.0%), HPV52 (10.3%), HPV33 (8.8%), and HPV18 (7.0%) were the dominant subtypes in the CIN2+ lesions (Fig. 3, Table S1). The prevalence of HPV16 was positively correlated with the severity of cervical lesions (30.7% in normal, 34.2% in CIN1, 63.4% in CIN2+, P_trend_ < 0.0001). Similar results were found for HPV33 (4.3% in normal, 6.4% in CIN1, 8.8% in CIN2+, P_trend_ < 0.0001). Distribution of multiple infections in women was as follows: 26.9% of multiple hrHPV infections in overall population (Fig. 3A), 25.3% in normal pathology, 32.2% in CIN1, and 29.3% in CIN2+, respectively (Fig. 3B-D). The proportions of each HPV type involved in multiple infections ranged from 1.0% for HPV11 to 12.6% for HPV16.

### Hierarchical classification for HPV genotypes and cumulative PPV/ sensitivity/ specificity for CIN2+/CIN3+

The risk determination of each genotype for detecting CIN2+ and CIN3+ was estimated by a hierarchical ranking of multiple infections, which resulted in similar hierarchies for CIN2+ and CIN3+. The overall ranking orders were HPV16, 33, 35, 58, 31, 68, 18, 56, 52, 66, 51, 59, 45 and 39 for CIN2+ (Table 2) and HPV16, 33, 31, 58, 45, 66, 52, 18, 35, 56, 51, 68, 59 and 39 for CIN3+ (Table 3). The PPV for CIN2+ was greatest for HPV16, being 27.4%. The PPV of HPV33 for CIN2+ was slightly lower at 26.0% univariately, and when multiple infections with HPV16 were excluded, it was 23.5%. Similar results were showed for CIN3+. HPV18 was ranked low in the 7th/8th place for CIN2+/CIN3+ respectively. Cumulative sensitivities and 1-specificities for CIN2+/CIN3+ as the number of hierarchical HPV types were sequentially increased are showed in Tables 2 and 3. The ROC curves for cumulative sensitivities and 1-specificities after adjusting for type hierarchy were plotted in Fig. S1, the areas under the ROC curves were 0.75 for CIN3+ and 0.71 for CIN2+ respectively.

**Table 2:** Cumulative PPV/Sensitivity/Specificity (%) for CIN2+ in the triage of hrHPV-positive women

hrHPV types	Primary type	For the new type		CIN2+/HPV+	Cumulative types		
	PPV (%)	CIN2+/HPV+	PPV (%)		PPV (%)	SEN (%)	SPE (%)
16	27.4	279/1020	27.4	279/1020	27.4	63.8	67.8
33	26.0	28/119	23.5	307/1139	27.0	70.3	63.8
35	17.6	10/59	16.9	317/1198	26.5	72.5	61.7
58	18.2	46/292	15.8	363/1490	24.4	83.1	51.0
31	16.8	18/127	14.2	381/1617	23.6	87.2	46.2
68	9.9	8/121	6.6	389/1738	22.4	89.0	41.3
18	8.3	16/265	6.0	405/2003	20.2	92.7	30.5
56	10.4	5/84	6.0	410/2087	19.6	93.8	27.0
52	11.7	15/270	5.6	425/2357	18.0	97.3	15.9
66	5.7	4/74	5.4	429/2431	17.6	98.2	12.9
51	7.2	5/131	3.8	434/2562	16.9	99.3	7.4
59	7.9	2/56	3.6	436/2618	16.7	99.8	5.0
45	10.3	1/32	3.1	437/2650	16.5	100.0	3.7
39	4.9	0/85	0.0	437/2735	16.0	100.0	0.0

*Abbreviations*: *CIN2+* cervical intraepithelial neoplasia 2 or worse, *SEN* sensitivity, *SPE* specificity, *PPV* positive predictive value

**Table 3:** Cumulative PPV/Sensitivity/Specificity (%) for CIN3+ in the triage of hrHPV-positive women

hrHPV types	Primary type	For the new type		Cumulative types			
	PPV (%)	CIN3+/HPV+	PPV (%)	CIN3+/HPV+	PPV (%)	SEN (%)	SPE (%)
16	18.0	184/1020	18.0	184/1020	18.0	73.3	66.3
33	15.3	17/119	14.3	201/1139	17.6	80.1	62.2
31	8.2	11/127	8.7	212/1266	16.7	84.5	57.6
58	8.5	21/292	7.2	233/1558	15.0	92.8	46.7
45	8.8	1/32	3.1	234/1590	14.7	93.2	45.4
66	2.8	2/74	2.7	236/1664	14.2	94.0	42.5
52	6.0	6/270	2.2	242/1934	12.5	96.4	31.9
18	3.5	5/265	1.9	247/2199	11.2	98.4	21.4
35	7.1	1/59	1.7	248/2258	11.0	98.8	19.1
56	4.5	1/84	1.2	249/2342	10.6	99.2	15.7
51	1.7	1/131	0.8	250/2473	10.2	99.6	10.5
68	4.0	1/121	0.8	251/2594	9.7	100.0	5.7
59	1.0	0/56	0.0	251/2650	9.5	100.0	3.4
39	2.2	0/85	0.0	251/2735	9.2	100.0	0.0

*Abbreviations*: *CIN3+* cervical intraepithelial neoplasia 3 or worse, *SEN* sensitivity, *SPE* specificity, *PPV* positive predictive value

### Relative risk of multiple infection vs. single infection for CIN2+

When multiple infections were present, hrHPV type with the highest PPV within the hierarchy was used for each woman, the risk of multiple infections versus single infections for CIN2+ was not statistically significant for women infected with HPV16 (OR = 0.8, 95%CI = 0.6-1.1, *p* = 0.144). After excluding individuals coinfected with types higher in the hierarchy, the odds of multiple infections versus single infections having CIN2+ were not statistically significant for all the other genotypes after conservative Bonferroni correction (*P* > 0.0036, Table 4). Furthermore, we didn’t observe a significant OR of CIN2+ according to multiple infections versus single infections when HPV16 was present, with ORs of 1.1 (95%CI = 0.8-1.4), 0.9 (95%CI = 0.6-1.4) and 1.0 (95%CI = 0.8-1.3) for women infected with 2, ≥3, and ≥ 2 genotypes, respectively. Similar results were found when HPV16 was not present (Table 5).

**Table 4:** Logistic regression analyses of Odds ratio (OR) for CIN2+ vs ≤ CIN1 according to multiple vs. single HPV infection

hrHPV types	Single infection	Multiple infection	OR (95% CI)^**†**^	***P***-value^**††**^
	No. CIN2+/ total	No. CIN2+/ total		
16	194/667	85/353	0.8 (0.6-1.1)	0.144
18	16/230	0/35	NA	NA
31	15/88	3/39	0.4 (0.1-1.4)	0.132
33	23/71	5/48	0.2 (0.1-0.6)	0.004
35	8/38	2/21	0.5 (0.1-3.2)	0.462
39	0/85	0/0	NA	NA
45	1/23	0/9	NA	NA
51	3/121	2/10	11.0 (1.5-80.6)	0.018
52	13/199	2/71	0.4 (0.1-1.9)	0.248
56	1/58	4/26	6.5 (0.4-99.4)	0.180
58	26/193	20/99	1.6 (0.8-3.1)	0.178
59	2/43	0/13	NA	NA
66	3/67	1/7	4.9 (0.4-64.6)	0.224
68	3/97	5/24	8.8 (1.8-43.8)	0.008

^†^The analysis was adjusted for age. ^††^*P*-values from multiple comparisons were corrected at a Bonferroni threshold (*P* = 0.05/14 = 0.0036)

*Abbreviations*: *CI* confidence interval, *CIN2+* cervical intraepithelial neoplasia 2 or worse, *OR* odds ratio, *NA* not available

**Table 5:** Multivariate logistic regression analyses of multiple infections and HPV vaccine subgroups

Characteristics	N of CIN2+ (%)	OR (95%CI)	***p***-value
**Vaccine subgroup**^**a**^			
16/18	295 (67.0)	4.6 (3.2-6.7)	<0.001
6/11^**b**^	3 (0.7)	NA	NA
31/33/45/52/58	108 (24.5)	2.5 (1.7-3.7)	<0.001
16/18/31/33/45/52/58	403 (91.3)	3.7 (2.6-5.3)	<0.001
35/39/51/56/59/66/68	34 (7.7)	1	
**Age (Y)**	440 (100.0)	0.99 (0.98-1.0)	0.086
**Screening sites**			
County A	135 (30.7)	0.6 (0.5-0.8)	< 0.001
County B	153 (34.8)	0.9 (0.7-1.1)	0.197
County C	152 (34.5)	1	
**HPV infection pattern**			
lrHPV only	3 (0.7)	NA	NA
hrHPV only	424 (96.4)	1.0 (0.5-2.0)	0.94
hrHPV+ lrHPV	13 (3.0)	1	
**No. HPV types**^**c**^			
1	311 (70.7)	1	1
2	99 (22.5)	1.1 (0.8-1.4)	0.602
≥ 3	30 (6.8)	0.9 (0.6-1.4)	0.568
≥ 2 (Multiple infection)	129 (29.3)	1.0 (0.8-1.3)	0.838
**No. types, non-HPV16**^**c**^			
1	117 (26.6)	1	
2	37 (8.4)	1.1 (0.8-1.7)	0.543
≥ 3	7 (1.6)	0.5 (0.2-1.2)	0.128

*Abbreviations*: *OR* odds ratio, *CI* confidence interval, *NA* not available, *CIN2+* cervical intraepithelial neoplasia 2 or worse

^**a**^The analysis was adjusted for age, screening sites

^**b**^HPV6/11 indicates only HPV6/11 infection, but excludes those coinfected with HPV6/11 and high-risk genotypes

^**c**^The analysis was adjusted for age, screening sites and HPV infection pattern

### Vaccine coverage and potential impact of vaccines

Table 3 shows the vaccine coverage of histologic abnormality assessed by the hierarchical or proportional attribution models. The hierarchical model showed that HPV6/11/16/18 covered by 4-valent vaccine potentially contributed to 57.7% of CIN2 lesions, 74.9% of CIN3 and 81.3% of cancers. While HPV6/11/16/18/31/33/45/52/58 covered by nonavalent vaccine was potentially responsible for 85.2% of CIN2 lesions, 97.9% of CIN3 and 93.8% of cancers. In addition, only 3 cases of CIN2 were attributed to lrHPV (Table 6). Similar results were found for the proportional model. Moreover, compared with HPV35/39/51/56/59/66/68 not covered by the nonavalent vaccine, HPV16/18 covered by all vaccines showed the highest risk for CIN2+, with a significant OR of 4.6 (95%CI = 3.2-6.7, *P* < 0.001), followed by HPV31/33/45/52/58 covered by the nonavalent vaccine with a significant OR of 2.5 (95%CI = 1.7-3.7, *P* < 0.001) (Table 5).

**Table 6:** Coverage of vaccine genotypes according to histological grades

Vaccine subgroups	Histology, n (%)				Total
	≤CIN1	CIN2	CIN3	Cancer	
**Hierarchical model**^**a**^					
6/11^**b**^	73 (3.1)	3 (1.6)	0 (0.0)	0 (0.0)	76
16/18	990 (49.8)	106 (56.1)	176 (74.9)	13 (81.3)	1285
31/33/45/52/58	732 (30.9)	52 (27.5)	54 (23.0)	2 (12.5)	840
35/39/51/56/59/66/68	576 (24.3)	28 (14.8)	5 (2.1)	1 (6.3)	610
6/11/16/18	1063 (44.8)	109 (57.7)	176 (74.9)	13 (81.3)	1361
6/11/16/18/31/33/45/52/58	1795 (75.7)	161 (85.2)	230 (97.9)	15 (93.8)	2201
**Proportional model**					
6/11^**b**^	73 (3.1)	3 (1.6)	0 (0.0)	0 (0.0)	76
16/18	904.3 (38.2)	93.5 (49.5)	172.4 (73.3)	14.6 (91.3)	1184.7
31/33/45/52/58	691.2 (29.2)	60.6 (32.1)	57.7 (24.6)	1.4 (8.8)	811.0
35/39/51/56/59/66/68	702.5 (29.7)	31.9 (16.9)	4.9 (2.1)	0 (0.0)	739.3
6/11/16/18	977.3 (41.3)	96.5 (51.1)	172.4 (73.3)	14.6 (91.3)	1260.7
6/11/16/18/31/33/45/52/58	1668.5 (70.5)	157.1 (83.1)	230.1 (97.9)	16.0 (100.0)	2071.7
Total	2371 (84.3)	189 (6.7)	235 (8.4)	16 (0.6)	2811

*Abbreviation*: *CIN* cervical intraepithelial neoplasia

^**a**^According to hierarchical ranking for CIN2+

^**b**^HPV6/11 indicates only HPV6/11 infection, but excludes those coinfected with HPV6/11 and high-risk genotypes. Moreover, HPV6/11 were not included into two attribution models

## Discussion

HPV testing on self-samples is easily centralized through a dry transport card, and centralization reduces the overall cost of the laboratory equipment [29]. This cross-sectional study was conducted efficiently in a large-scale population and consumed only about half a year by using Seq HPV assay owing to its high-throughput capacity, high sensitivity, and low cost per case [24]. The successful implementation of cervical screening program based on HPV testing on self-samples provides a crucial guidance for the prevention and control of cervical cancer particularly in low-resource areas.

Due to the risk variation of different genotypes, information on cervical lesions conferred by specific genotypes is helpful for optimizing genotype-based screening strategy [12, 30]. However, the existence of multiple infections complicated type-specific risk assessment. In the current study, ranking of HPV types by PPVs provided similar hierarchies for CIN2+ and CIN3+, with HPV16/33 posing the greatest risk. Cuzick et al. reported a ranking based on PPVs for CIN3+ with HPV16/33 to be the highest ranks in a referral population [31]. Adcock et al. confirmed HPV16, 33, and 31 posing the greatest risks for precancers [6]. Notably, the risks of CIN2+/CIN3+ among women infected HPV18 were ranked low in the 7th/8th place. This is somewhat surprising but in line with mounting evidences [6, 32]. Despite the low risk of HPV18 in the study, detection of HPV18 in cervical cancers is second only to HPV16 in prior studies [13, 33].

To date, HPV16/18 genotyping has been well established as a triage tool for HPV-positive women in guidelines [7]. However, due to the low sensitivity of HPV16/18 as a triage relative to cytology triage [34], there is still an uncertainty regarding the extent to which adding hrHPV genotypes beyond HPV16/18 into the triage could enhance disease detection. Moreover, deciding which types to include in a triage strategy must weigh the absolute risk of cervical disease related to genotypes [35]. Both HPV31 and HPV58 ranked high for CIN2+/CIN3+, which is consistent with prior studies [36, 37]. Notably, detection of a specific genotype predicts risk of precancers, but cannot differentiate between a transient infection and detectable lesions [35]. Thus genotyping alone might not be accuracy enough to be the sole triage test [34], and the AUCs of genotypes were only 0.71 for CIN2+ and 0.75 for CIN3+. However, when present, HPV33/31/58 may be given a priority when deciding upon the need for immediate colposcopy similarly to HPV16, which reduces the follow-up burden. In addition, HPV39/59/51, ranked low both for CIN2+/CIN3+, may be considered as ‘intermediate risk’ types, which was similar to prior studies [6, 11, 12]. When present, HPV39/59/51 types, which did not appreciably contribute to relevant cervical lesions, might be permitted follow-up in 1 year with the expectation of viral clearance. For the remaining hrHPV types, information of other tests such as LBC or p16 immunocytology may be obtained for referring.

The findings above support the crucial role of extended genotyping in cervical screening [8], and provide evidence for the development of new technology for the detection of HPV types. In addition, this study was conducted at three counties in Henan Province, China with a shortage of cytologists. HPV genotypes are obtained automatically with HPV results, thus particularly useful for the settings where lack cytology results such as HPV-based screening on self-samples or at rural areas. Moreover, when the information on separate genotype is identified, more detailed management and appropriate follow-up strategies can be established at an earlier time for individuals according to genotypes.

In this study, multiple HPV infections were common with 26.9% found in overall study population. Numerous previous studies have reported similar results, ranging from 11.4 to 40.0% [6, 10, 32, 36]. Multiple HPV infection is attributed to certain factors, such as age, smoking, sexually activity, lifetime number of sexual partners, and immunodeficiency [10, 36]. Nevertheless, currently the impact of multiple infection on the risk of cervical lesions has not been established yet. Whether these infections occur by chance or as a result of interactions between HPV genotypes is still conflicting [10, 15]. Herrero et al. showed that co-infections may be associated with HPV persistence, and increase the duration of infection and the risk of cervical diseases [38]. Still overwhelming studies showed no impact [6, 15, 39]. Recently Iacobone et al. confirmed that HPV coinfections were significantly associated with lower risk of CIN2+, whereas single infections were more likely in cervical cancers and precancers [40]. Another study tested hrHPV by Cobas4800 assay showed that HPV16 co-infected with other types appeared to have a lower risk of CIN3+ than single HPV16 infection [16]. Although Cobas4800 assay has the ability to detect 14 types of hrHPV, it is impossible to distinguish the separate types except for HPV16/18. Therefore, hrHPV coinfection has not been fully and adequately explored, especially among the pool 12 types of hrHPV.

The current study, using a full genotyping assay-Seq HPV assay, revealed that women infected with HPV16 only had no significantly different risk for CIN2+ than those co-infected with HPV16 and other types. Similar results were found for the other genotypes excluding HPV16. However, data regarding the risk of CIN2+ associated with other genotypes excluding HPV16 should be interpreted with caution due to the either relatively low prevalence or the limited number of CIN2+ cases. Interestingly, the inclusion of coinfection with HPV types lower in the hierarchy added little to the risk prediction for CIN2+, possibly due to the fact that genotype with the highest PPV largely determines the risk in multiple infections and the impact of the additional genotypes is small [6]. Likewise, generally having a multiple infection conferred no additional risk for single HPV infection both in the presence or absence of HPV16, which was in accordance with a prior study [39]. But these findings must be interpreted cautiously and further confirmed via longitudinal studies, and the potential mechanisms warrant further investigation.

Updated evidence on coverage and carcinogenesis of vaccination genotypes is also essential for assessing potential impacts of HPV vaccines [13, 36], particularly in China prior to a National Immunization Program. In this study, HPV16/58/52/33/18/31 were the dominant genotypes in cervical precancers or cancers, which was consistent with prior studies [12, 41]. Moreover, hrHPV types covered by the nonavalent vaccine were associated with significantly higher risk for CIN2+ than hrHPV types not covered by the vaccine. Fortunately, similar to a worldwide study [42], most cervical cancers were potentially responsible for nonavalent vaccine in the study population. Notably, addition of HPV 6/11 did add only 3 cases of CIN2 but no CIN3+, hence cervical cancer screening may not include testing for lrHPV types. However, vaccine targeted HPV 6/11 prevents most of external genital wart cases [17].

Our findings may help healthcare authorities assess the impact of vaccination programs, providing a basis for the application of tailored HPV vaccines in Central China. Quadrivalent HPV vaccination was associated with a substantially reduced risk of invasive cervical cancer [43]. Huh et.al. reported that the nonavalent vaccine showed efficacy against cervical lesions related to HPV31/33/45/52/58 and similar efficacy toward HPV 6/11/16/18 as the 4-valent vaccine [17]. If our estimations are true, and high coverage vaccination can be implemented quickly, combined with the low proportion of cervical diseases and low risk of HPV types not covered by the nonavalent vaccine in the current study, vaccine intervention would achieve a great effect on prevention and eventual elimination of cervical cancer.

There are several limitations of this study. Firstly, the study population may not represent the general screening population. The selection of HPV-positive women who were referred for colposcopy was based on sequential indicators-HPV16/18, VIA, and p16 staining, not randomly, and management guidelines were not always followed by screen-positive women exactly. Additionally, there were no measures to check CIN2+ among those with HPV-negative results, thus the false negative rate is unknown. These facts reflecting a real-life situation in routine cervical screening programs rather than in a clinical trial. Another caveat is that HPV distribution according to cytological abnormalities wasn’t added since LBC was conducted on the p16 preservative liquid, which hasn’t been validated clinically yet; However, the association between genotypes and histologic abnormalities is more meaningful. In addition, we acknowledge that a cross-sectional data has limited power to predict the role of genotypes and multiple infections on disease progression or regression. Actually, the baseline disease detection in this study was comparable to what was detected in a longitudinal study [6]. Finally, the hierarchical and proportional attribution models used may not completely match the true causal assignment due to two major drawbacks [32]. First, they assume that every woman has a single lesion. Second, they may overestimate the effect of vaccination genotypes that are relatively common in the general population and coincidentally detected in lesions [27].

The strength of this study lies in the large sample size from a well-organized, population-based cervical screening program which ensures the strong statistical strength and enhances the suitability. Moreover, all enrolled women had a definite histologic diagnosis through colposcopy-directed biopsy, and the slides were verified by senior gynecological pathologists from PUSH, which ensures the accuracy and reliability of the outcomes. Histology diagnoses are well accepted as the gold standard for cervical diagnosis and best endpoint which could gain great implications in clinical practice [16]. Furthermore, primary HPV screening was completed at about 1 month, which eliminates the impact of time span on HPV prevalence. In addition, due to the prospective nature of this study, the missing results of Seq HPV assay or histology were minimized.

## Conclusions

Genotyping by Seq HPV assay was valuable in improving risk stratification of HPV-positive self-samples, with HPV33/31/58 types ranked high risk and HPV39/59/51 types ranked low risk both for CIN2+/CIN3+. Coinfection with HPV types lower in the hierarchy conferred little to the risk for CIN2+ associated with single hrHPV infection. Moreover, incidence rates of cervical cancer and precancer were substantial attributable to HPV types covered by nonavalent vaccine. This study provides critical insights into vaccine strategies, and establishes the foundation for the development of genotype-specific screening approaches on self-samples, which is particularly useful for cervical screening in rural settings.

## Supplementary Information

**Additional file 1.**

## Data Availability

The data that support the findings of this study are included in the manuscript, or available on request from the corresponding author.

## Abbreviations

CI: Confidence interval
CIN: Cervical intraepithelial neoplasia
CIN2+/CIN3 +: CIN 2/3 or worse
HPV: Human papillomavirus
hrHPV: High-risk human papillomavirus
LBC: Liquid-based cytology
OR: Odds ratio
PCR: Polymerase chain reaction
PPV: Positive Predictive Value
PUSH: Peking University Shenzhen Hospital
ROC: Receiver operator characteristic
HSIL: High-grade Squamous Intraepithelial Lesion
FTA: Flinders Technology Associates
