# Supplementary material for: Roles of extended human papillomavirus genotyping and multiple infections in early detection of cervical precancer and cancer and HPV vaccination
Source: BMC Cancer. 2022 Jan 6;22:42. doi: 10.1186/s12885-021-09126-3 (PMC8734293; doi:10.1186/s12885-021-09126-3)
Supplement: Supplementary file 1 — Additional file 1. [file 12885_2021_9126_MOESM1_ESM.docx]

**Roles of extended human papillomavirus genotyping and multiple infections in early detection of cervical precancer and cancer and HPV vaccination**

Fangbin Song^1,2#^, Peisha Yan^1,2#^, Xia Huang^1,2^, Chun Wang^1,2^, Hui Du^1,2*^, Xinfeng Qu^3*^, and Ruifang Wu^1,2*^


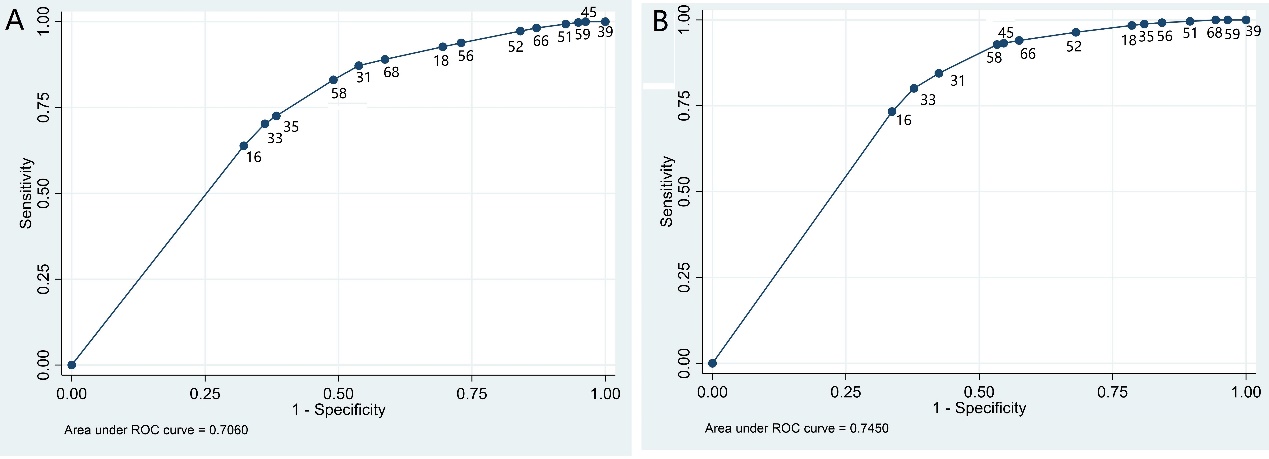


**FIGURE S1** Receiver operating characteristic **(**ROC) curves of cumulative sensitivity/1-specificity of hierarchical and ordering hrHPV genotypes **(A)** for CIN2+, area under curve (AUC) = 0.71 (0.68-0.73); **(B)** for CIN3+, AUC= 0.75 (0.72-0.77).

**Table S1** Individual genotypes by histology

| HPV genotypes |  | Histology, n (%) | | |
| --- | --- | --- | --- | --- |
|  | Total | ≤CIN1 | CIN2 | CIN3+ |
| 6^†^ | 115 | 83 (72.2) | 20 (17.4) | 12 (10.4) |
| 11^†^ | 47 | 32 (68.1) | 11 (23.4) | 4 (8.5) |
| 16 | 1,020 | 602 (59.0) | 139 (13.6) | 279 (27.4) |
| 18 | 375 | 290 (77.3) | 54 (14.4) | 31 (8.3) |
| 31 | 184 | 134 (72.8) | 19 (10.3) | 31 (16.8) |
| 33 | 150 | 85 (56.7) | 26 (17.3) | 39 (26.0) |
| 35 | 85 | 54 (63.5) | 16 (18.8) | 5 (5.9) |
| 39 | 182 | 144 (79.1) | 29 (15.9) | 9 (4.9) |
| 45 | 68 | 52 (76.5) | 9 (13.2) | 7 (10.3) |
| 51 | 236 | 186 (78.8) | 33 (14.0) | 17 (7.2) |
| 52 | 386 | 278 (72.0) | 63 (16.3) | 45 (11.7) |
| 56 | 134 | 101 (75.4) | 19 (14.2) | 14 (10.4) |
| 58 | 363 | 235 (64.7) | 62 (17.1) | 66 (18.2) |
| 59 | 101 | 78 (77.2) | 15 (14.9) | 8 (7.9) |
| 66 | 141 | 108 (76.6) | 25 (17.7) | 8 (5.7) |
| 68 | 202 | 147 (72.8) | 35 (17.3) | 20 (9.9) |

Note: ^†^HPV6 infection incudes single HPV6 infection, and co-infection with HPV6 and high-risk genotypes and/or HPV11; HPV11 infection incudes single HPV11 infection, and co-infection with HPV11 and high-risk genotypes and/or HPV6.
